# Beyond replacement anxiety: a psychological framework for understanding AI in natural science research

**DOI:** 10.3389/fpsyg.2026.1824256

**Published:** 2026-04-16

**Authors:** Xinyi Qi, Yuchen Gao, Peiqing Sun

**Affiliations:** 1 Department of Basic Teaching, Chuzhou Polytechnic, Chuzhou, China; 2 School of Management, Chuzhou Polytechnic, Chuzhou, China; 3 School of Chemistry and Chemical Engineering, Nanjing University, Nanjing, China

**Keywords:** artificial intelligence, institutional climate, psychological adaptation, researcher identity, scientific labor

## Abstract

Artificial intelligence (AI) is moving from a peripheral aid to a structuring force in natural science research. Large language models, multimodal systems, autonomous laboratories, and agentic tools now assist with literature analysis, coding, image interpretation, experimental optimization, manuscript preparation, and elements of peer review. Public discussion has largely emphasized acceleration, automation, and discovery potential. Yet the psychological implications of this transition for researchers remain underdeveloped. This perspective argues that AI in natural science should be understood not only as a technical shift, but also as a reorganization of the psychological environment of scientific work. Drawing on recent work on autonomous experimentation, AI-assisted writing and review, technostress, AI workplace anxiety, attitudes toward AI at work, and human–AI delegation, we propose a four-part framework organized around labor visibility, identity stability, accountability under delegated cognition, and institutional climate. We treat this framework as a theoretically grounded and partly inferential lens whose expression is expected to vary across disciplines, tasks, career stages, and institutional settings. We further argue that responsible AI adoption in science should be judged not only by speed or scale, but also by whether research systems preserve human agency, interpretive responsibility, developmental learning, and sustainable scientific careers. A psychological perspective does not oppose scientific modernization; rather, it clarifies the human conditions under which AI-enabled science can remain rigorous, trustworthy, and professionally livable.

## Introduction

Artificial intelligence has moved rapidly toward the operational center of natural science research. What began as specialized support for data-rich tasks now shapes literature discovery, coding, image analysis, materials screening, autonomous experimentation, scientific writing, and scholarly evaluation. In recent years, autonomous and self-driving laboratory systems have demonstrated that robotics, machine learning, optimization, and feedback can be integrated into closed-loop research workflows across materials science and biotechnology (Szymanski et al., 2023; Canty et al., 2025; Fushimi et al., 2025; Singh et al., 2025). At the same time, generative AI tools are

increasingly used to summarize literature, organize arguments, draft prose, generate code, and support review-related work (Kharlamova and Stavytskyy, 2024; Cohen and Moher, 2025; Hysaj et al., 2025). AI is therefore acting not only on the production of science, but also on parts of the communicative and evaluative infrastructure through which science is judged.

This transition is often narrated through the language of efficiency and progress. Across discussions of self-driving laboratories, AI-assisted scientific writing, and AI-enabled research workflows, authors have argued that AI can accelerate discovery, reduce routine burden, broaden access to advanced analytical capability, and expand the range of problems that a single researcher or team can address (Pyzer-Knapp et al., 2022; Abolhasani and Kumacheva, 2023; Zhang et al., 2025). These claims are often justified. Yet they remain incomplete. They tell us much about what research systems may gain and much less about what researchers experience. Scientific work is not merely a chain of technical operations. It is also developmental work, identity work, and moral work. Researchers build expertise through repeated practice, attach meaning to authorship and judgment, and depend on communities of recognition to validate competence and trustworthiness.

For this reason, AI in natural science should be treated as a psychological transition as much as a technological one. When tools perform tasks historically associated with expertise formation and scholarly contribution, they also change how researchers perceive their own value, how accountability is distributed, and how institutional expectations are interpreted. Research from organizational psychology and human–AI collaboration shows that AI-related job anxiety can undermine wellbeing, that AI transformation can intensify job insecurity even while stimulating adaptive behavior, and that AI use may generate new forms of technostress unless governance and support are clear (Brougham and Haar, 2018; Tarafdar et al., 2019; Bankins et al., 2024; Park et al., 2024; Feng et al., 2025; Högemann et al., 2025; Sha and Chai, 2025). Although much of this evidence comes from workplaces beyond academia, it illuminates mechanisms that are increasingly relevant inside laboratories, research groups, and scientific institutions. Accordingly, the present framework should be read not as a claim that all of these dynamics have already been fully established across natural science settings, but as a theoretically reasoned account of pressures that are becoming plausible, visible, and testable in AI-rich research environments.

This perspective argues that the psychological significance of AI-enabled science can be clarified through four analytically distinct but interrelated dimensions: labor visibility, identity stability, accountability under delegated cognition, and institutional climate. Labor visibility concerns whether human effort remains recognizable in AI-mediated workflows; identity stability concerns the continuity of professional self-concept and developmental learning; accountability under delegated cognition concerns the asymmetry between delegated cognitive operations and retained human responsibility; and institutional climate concerns the local norms and governance structures that shape how the other three dimensions are interpreted and managed. Together, these dimensions help explain why AI can feel simultaneously empowering and destabilizing in research environments. The central claim of this article is that the future of science should not be evaluated only by what AI can do, but also by what kinds of researchers, research practices, and research lives AI-rich systems make possible.

## Why these psychological dimensions matter now

The urgency of a psychological lens is increased by the speed and opacity of AI diffusion in science. Individual researchers are often expected to experiment with tools before disciplinary standards have stabilized. This produces a temporal mismatch: adoption is fast, but norms are slow. In such conditions, uncertainty itself becomes a burden. Researchers must not only learn new systems, but also infer which uses will later be judged skillful, careless, ethical, or professionally compromising. That burden is especially heavy in competitive environments where grants, publication timelines, and employability already depend on subtle signals of competence. At the same time, these pressures are unlikely to be uniform. AI adoption differs across laboratory sciences, computational fields, interdisciplinary teams, and publication-centered tasks, so the framework proposed here is intended as a cross-context lens rather than as a claim of identical effects across all natural science domains.

A psychological perspective is also necessary because scientific training is cumulative and developmental. The path from student to independent researcher depends on repeated exposure to tasks that are sometimes inefficient but formative: reading broadly, writing imperfect drafts, debugging manually, testing interpretations against resistant evidence, and learning how not to overstate conclusions. If AI compresses these stages without redesigning mentorship and evaluation, institutions may preserve output while weakening the conditions under which expert judgment is formed. The issue is therefore not whether AI should be used, but whether the surrounding culture can distinguish between productive augmentation and the quiet erosion of scientific development.

This concern is sharpened by the growing visibility of AI in scientific writing itself. Recent evidence suggests that large language model use is already detectable across a substantial volume of scientific papers, with rapid growth in some fields and publication contexts (Liang et al., 2025). As AI becomes more common in drafting, polishing, and reviewing scientific text, researchers must navigate not only technical usefulness but also questions of authenticity, disclosure, calibration of trust, and reputational risk (Al-Hashimi, 2026).

## AI is changing the architecture of scientific work

The scientific significance of AI can no longer be reduced to text generation or to general-purpose assistive tools such as large language model chatbots, code assistants, and agentic research interfaces. Autonomous laboratory platforms already integrate culturing, experimental measurement, data analysis, and iterative hypothesis refinement in biotechnology and materials science (Li et al., 2025). In parallel, researchers increasingly rely on generative systems for literature exploration, coding support, figure interpretation, manuscript drafting, and reviewer assistance (Khalifa, 2023). This matters psychologically because the symbolic markers of scientific competence are becoming unstable. In earlier research cultures, being a strong scientist often meant demonstrating depth, patience, interpretive reliability, and the ability to work slowly through uncertainty. Those qualities still matter, but they are increasingly joined by expectations of AI fluency, rapid synthesis, and visible adaptability to machine-augmented workflows.

This shift should also be understood in historical context. Scientific work has long been reorganized by institutional and technical changes, including the expansion of publication metrics and “publish or perish” pressures, the rise of computational and programming skills as baseline expectations, and earlier waves of digitalization and automation (Aragón, 2013; Maer-Matei et al., 2019; Hong et al., 2025). In that sense, AI is not the first force to alter what counts as competence or productivity in research. What makes the current moment distinctive is that AI reaches beyond instrumentation or calculation and enters domains that are closely tied to professional judgment and self-concept, including reading, writing, coding, interpretation, and preliminary evaluation (Khalifa and Albadawy, 2024). The change is therefore not simply that scientists must learn another tool; it is that the boundary between performing expertise and supervising machine-generated intellectual work is being redrawn inside core scholarly tasks.

Importantly, AI does not simply replace human effort; it also redistributes it. As Autor (2015) argued the broader context of automation, technological change often alters task composition rather than straightforwardly eliminating work. In science, this means that visible production may become faster while invisible supervision, verification, and boundary-setting become more demanding. The result is not a simple subtraction of labor but a reorganization of where labor occurs, how it is valued, and how it is experienced.

## A psychological framework for AI-enabled science

**Figure 1:**
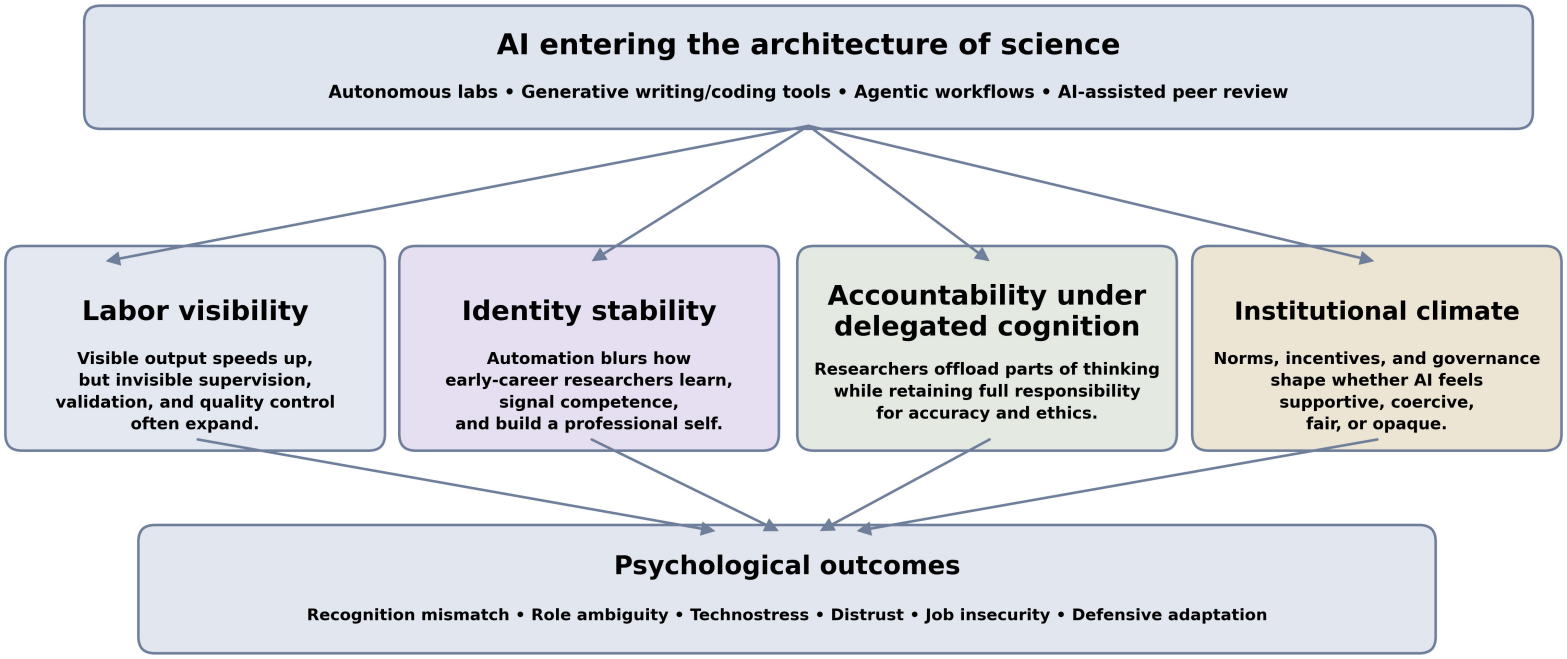
Conceptual overview of the four psychological dimensions through which AI reorganizes the experience of natural science research.

Figure 1 synthesizes the argument by showing how AI enters the architecture of science through autonomous laboratories, generative writing and coding tools, agentic workflows, and AI-assisted peer review. It organizes the article around four linked psychological dimensions—labor visibility, identity stability, accountability under delegated cognition, and institutional climate—thereby explaining why AI can be experienced as both enabling and destabilizing. A compact summary of these dimensions is provided in Supplementary Table S1 (see Supplementary material).

## Labor visibility

The first psychological dimension is labor visibility. Scientific work includes many forms of effort that are intellectually demanding but weakly visible in final outputs: reading conflicting studies, cleaning unusable data, debugging fragile code, identifying subtle artifacts, checking whether an elegant answer is actually wrong, and deciding when not to trust an apparently plausible result. AI can make outputs appear faster and smoother, but it does not remove the need for judgment. Instead, it often relocates judgment into a less visible layer of work: validation, provenance checking, calibration of trust, error detection, and epistemic quality control.

This shift matters because institutions tend to reward what is easiest to measure. A polished literature synthesis produced in minutes does not erase the expertise required; rather, it can make the underlying distribution of effort harder to see by shifting a larger share of the human contribution toward checking, selecting, rejecting, and contextualizing machine-generated material. In this sense, the concern is not that AI obscures expertise as such, but that it may obscure where expertise is now being exercised (Ribeiro et al., 2023). For example, an AI system may rapidly assemble a coherent review of a fast-moving topic, yet the researcher must still decide whether key studies were omitted, whether the cited evidence is being interpreted in the right disciplinary context, whether the narrative overstates consensus, and whether confident prose is masking epistemic weakness (Steyvers et al., 2025). Code assistance may accelerate prototyping while concealing the labor needed to inspect assumptions and failure modes. Autonomous experimentation may compress visible trial-and-error while increasing the importance of data stewardship, constraint design, and interpretive restraint (Casukhela et al., 2022; Ali et al., 2025). In this sense, AI may reduce the visibility of human scientific labor precisely where judgment remains most important.

The psychological consequence is a potential recognition mismatch. Researchers may continue working intensely, but in ways that become less legible to supervisors, committees, and institutions. Over time, this weakens the perceived link between effort and esteem, and can foster fatigue, cynicism, or a sense of disposability. The issue is especially acute for early-career scientists because many foundational abilities are built through the very tasks that AI now appears to streamline. A psychologically informed adoption of AI, therefore, asks not only how much time is saved, but also which forms of expertise are displaced from view and which forms of human contribution are no longer rewarded clearly.

## Identity stability

The second dimension is identity stability. Research is not only the production of knowledge; it is also the formation of a professional self. Scientists become careful readers, skeptical interpreters, resourceful coders, and responsible authors through repeated cycles of practice and recognition. AI can unsettle this process when it performs tasks that previously served as markers of emerging competence. AI is also distinctive from earlier research technologies because it acts not only on the material or computational environment of science, but also on symbolic activities through which the professional self is formed. Earlier tools often changed how data were collected, processed, or modeled; generative and agentic AI also intervenes in drafting, summarizing, coding, explaining, and reviewing—activities that many researchers experience as evidence of their own intellectual authorship (Hu et al., 2025). For that reason, AI can alter not only workflow efficiency but also the felt basis on which professional identity is built.

This issue may be particularly sharp for junior researchers. Literature synthesis, exploratory coding, first-draft writing, figure preparation, and preliminary analysis have long functioned as spaces in which young scientists both learned and demonstrated value. When these domains become partially automated, the route from effort to identity may become less clear. The concern here is theoretically grounded rather than conclusively established for all research settings: if work that once served as a developmental site is increasingly delegated, the formation of competence, confidence, and professional self-understanding may become more fragile.

Recent studies suggest that people's reactions to AI at work are multidimensional, incorporating not only perceptions of utility and quality, but also anxiety, job insecurity, and perceived humanlikeness (van den Broek, 2025; Yadav et al., 2026). In academic settings, fluency with AI is also becoming a reputational signal. Researchers may therefore feel pressure to perform enthusiastic adoption even when their own experience is ambivalent. This can generate identity dissonance: a scientist may publicly embrace AI as the language of progress while privately worrying that dependence on it weakens originality, authorship, or autonomy. At present, these identity implications should be read as plausible extensions requiring direct empirical examination in research-training environments.

The point is not that AI necessarily destroys scientific identity. Rather, identity becomes less stable when institutional symbols and lived developmental experience begin to diverge. If institutions continue to reward polished outputs without clarifying what counts as meaningful human contribution, they risk producing scientists who are outwardly productive but inwardly uncertain about the basis of their professional value.

## Accountability under delegated cognition

The third dimension is accountability under delegated cognition. AI allows researchers to offload portions of searching, drafting, coding, pattern recognition, and decision support. Delegation can be rational and beneficial. Yet responsibility for scientific integrity remains human. Authors, reviewers, principal investigators, and institutions still bear accountability for error, confidentiality, provenance, interpretation, and ethical judgment.

This asymmetry creates a distinctive psychological burden. When part of the cognitive process is delegated, but full responsibility for the result remains with the user, the task is not uniformly made easier. Instead, it often changes the human role into one centered on supervisory cognition: monitoring outputs, interrogating their basis, deciding when to rely on them, and determining when intervention or rejection is required (Elish, 2025). Researchers must ask whether an output is useful, whether it is trustworthy, whether it contains fabrication or distortion, whether its origin can be defensibly disclosed, and whether reliance on it could produce reputational harm. In this sense, AI often creates supervisory cognition rather than pure cognitive relief.

Research on human–AI advice-taking and productive delegation is relevant here. Effective collaboration depends less on blind acceptance than on calibration, selective reliance, and well-defined boundaries (Dietvorst et al., 2018; Fügener et al., 2022). At the same time, studies of LLM calibration show that people often overestimate the reliability of fluent machine outputs, especially when explanations sound confident or elaborated (Steyvers et al., 2025). This combination is especially risky in science, where linguistic polish can be mistaken for epistemic soundness.

Accountability concerns extend beyond individual cognition into scientific ethics and governance. Questions about disclosure, confidentiality in peer review, data sensitivity, and acceptable use cannot be resolved by technical convenience alone. Qualitative work on AI research ethics further suggests that ethical dilemmas emerge not only from model capability but from the institutional arrangements in which those capabilities are used (Jeon et al., 2025). Scientific work has always involved monitoring how knowledge is produced, but AI intensifies this reflexive burden by making the production process itself more opaque, distributed, and partly externalized to systems whose internal operations, training data, or provenance may be difficult for end users to inspect directly (Bommasani et al., 2024). For natural science researchers, the psychological result is a persistent form of boundary management: they are not only doing science, but continuously monitoring the legitimacy of how that science is being done.

## Institutional climate

The fourth dimension is institutional climate. AI adoption is never experienced in a vacuum. Its psychological effects are shaped by local norms, incentive structures, governance clarity, and informal expectations. In supportive environments, AI may be framed as a tool for reducing drudgery, widening access, and redirecting effort toward more meaningful reasoning. In competitive environments, the same tools may become benchmarks against which researchers are measured, quietly raising expectations while leaving uncertainty and responsibility intact. Institutional climate is therefore treated here as a contextual condition that shapes the meaning of the other three dimensions, rather than as a duplicate explanation of them.

A growing body of research supports this duality. Organizational studies show that AI is experienced by workers as both an opportunity and a source of strain, depending on context, implementation, and the distribution of control (Hornikel et al., 2021; Greaves and Colucci, 2025). AI workplace anxiety has been linked to reduced life satisfaction through negative emotions (Feng et al., 2025). Digital-AI transformation may also heighten job insecurity even where it triggers adaptive behaviors such as job crafting (Sha and Chai, 2025). Qualitative evidence on generative AI and technostress similarly shows that these tools often change work less by eliminating it than by shifting it toward monitoring, verification, and constant adaptation (Chang et al., 2024).

For science, this means that AI can feel simultaneously enabling and threatening. Researchers may appreciate assistance while suspecting that institutions will simply raise output expectations again. They may adopt AI to remain competitive while resenting the silent norm that one must always be faster, more fluent, and more adaptively available to new AI-mediated workflows. Here, “availability” refers less to literal 24-h connectivity than to the expectation that researchers should remain continuously responsive to new toolchains, monitor AI-supported processes, and update their practices whenever the technological baseline shifts (Kukulska-Hulme, 2012). If AI use in writing, analysis, and evaluation remains ambiguous, scientific culture may gradually move from collegial trust toward low-grade defensive vigilance. Researchers begin to wonder whether peers, reviewers, or competitors are being judged by the same standards, or whether hidden automation has become an unspoken advantage. Once such questions become routine, the emotional infrastructure of science becomes more fragile.

## From replacement anxiety to informed agency

**Figure 2:**
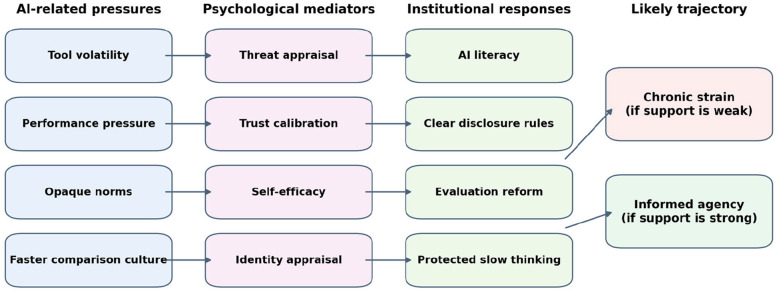
Proposed pathway from AI-related pressures in scientific work to either chronic strain or informed agency.

Figure 2 translates the framework into a process model. It shows how AI-related pressures in scientific work can accumulate into chronic strain when they are not moderated, but can be redirected toward informed agency when institutions provide AI literacy, transparent disclosure norms, mentorship, and evaluation reform.

Public discussion often frames AI in science through an overly simple binary: either AI will replace researchers, or it will harmlessly assist them. Neither position is adequate. AI does not need to eliminate whole occupations to alter the emotional reality of research. It can do so by changing recognition systems, competence signals, developmental pathways, and institutional expectations of constant adaptability.

At the same time, adaptation should not be romanticized. Some researchers will convert AI into a source of leverage, creativity, and better work design. But it would be ethically misguided to treat chronic insecurity as a desirable engine of modernization. A sustainable scientific profession cannot rely on anxiety as its default motivational structure (Jeffrey and Matakos, 2024).

A more constructive alternative is informed agency. Researchers need support that helps them move from diffuse threat to calibrated control. This includes AI literacy, but in a broader sense than tool proficiency alone. It involves understanding capability limits, hallucination risks, uncertainty communication, domain mismatch, privacy constraints, disclosure norms, and the psychological traps of both overtrust and undertrust. It also requires preserving the legitimacy of selective, critical, and even minimal use. A healthy scientific culture should not equate sophistication with maximal adoption. Instead, it should create space for reasoned judgment about when AI genuinely strengthens rigor, when it merely accelerates low-value output, and when it introduces unacceptable epistemic or ethical risk.

## Discussion

AI is changing natural science at the levels of method, workflow, communication, and evaluation. Its deeper significance, however, lies in how it reorganizes the psychology of being a researcher. It changes what labor is visible, what expertise feels stable, how accountability is experienced, and how institutions distribute trust and pressure. If these dimensions are ignored, scientific progress may come with an unacknowledged emotional and developmental cost. At the same time, the argument advanced here is not that all natural science fields are affected in the same way. The framework is expected to vary by discipline, career stage, task ecology, and institutional setting, and should therefore be treated as a structured lens for comparison rather than as a universal claim of uniform impact.

The challenge is therefore not to defend a pre-AI past or to surrender to automation as an unquestioned future. It is to design research cultures in which human agency remains meaningful inside AI-rich environments. This requires interventions that map directly onto the four dimensions identified above. For labor visibility, institutions should recognize verification, supervision, and epistemic quality-control work rather than rewarding only fast, visible outputs. For identity stability, mentorship and training systems should preserve developmental opportunities in reading, drafting, coding, and interpretation rather than treating all friction as inefficiency. For accountability under delegated cognition, AI literacy should be treated as a collective support system that includes trust calibration, disclosure practice, and boundary setting rather than as an individual self-defense project. For institutional climate, policies on authorship, disclosure, peer-review assistance, and data governance should be explicit enough to reduce moral fatigue and boundary-guessing.

The framework also invites a clearer empirical agenda. Future work could examine these dynamics at multiple levels, including individual researchers, research groups, doctoral and postdoctoral training environments, and institutional policy regimes. Candidate variables include perceived labor recognition, invisibility of validation work, identity insecurity, developmental role ambiguity, delegation burden, trust calibration, disclosure uncertainty, and perceived institutional permission to use AI cautiously or selectively. Mixed-methods designs that combine surveys, interviews, comparative institutional analysis, and field-based qualitative inquiry may be especially useful for testing where the framework travels well and where its boundary conditions become visible. A psychological perspective does not slow innovation; it makes innovation more sustainable. With such a perspective, we can ask not only what kinds of discoveries AI may enable, but also what kinds of researchers—and what kinds of research lives—scientific institutions will continue to make possible.

## Data Availability

The original contributions presented in the study are included in the article/Supplementary material, further inquiries can be directed to the corresponding authors.
