# Supplementary material for: Beyond replacement anxiety: a psychological framework for understanding AI in natural science research
Source: Front Psychol. 2026 Apr 16;17:1824256. doi: 10.3389/fpsyg.2026.1824256 (PMC13128544; doi:10.3389/fpsyg.2026.1824256)
Supplement: Supplementary file 1 [file Data_Sheet_1.pdf]

Supplementary Material

Dimension	Core mechanism	Likely indicators	Exemplar settings
Labor visibility	Judgment-intensive work becomes less visible even when it remains necessary.	Perceived recognition of checking work; visibility of validation labor; mismatch between output speed and acknowledged effort.	AI-assisted literature synthesis, code generation, automated data pipelines, and autonomous experiments.
Identity stability	Developmental tasks are partially delegated, potentially weakening the link between effort, learning, and professional self-understanding.	Identity insecurity, role ambiguity, perceived loss of developmental practice, and uncertainty about meaningful contribution.	Doctoral training, postdoctoral work, first-author drafting, exploratory analysis.
Accountability under delegated cognition	Cognitive operations can be offloaded while responsibility for accuracy, interpretation, and ethics remains human.	Delegation burden; trust calibration; disclosure uncertainty; perceived responsibility asymmetry.	AI drafting, coding assistance, decision support, and review-related tasks.
Institutional climate	Local norms, incentives, and governance shape whether AI is experienced as support, pressure, permission, or threat.	Governance clarity; perceived pressure to adopt; perceived permission for cautious or selective use; fairness perceptions.	Labs, departments, journals, funding, and evaluation systems.

Supplementary Table S1. Analytical distinction, core mechanism, likely indicators, and exemplar settings for the four psychological dimensions. Table S1 summarizes the four dimensions in compact form. The dimensions are related but not interchangeable: labor visibility focuses on whether effort remains recognizable; identity stability focuses on whether developmental learning continues to support a coherent professional self; accountability under delegated cognition focuses on the retention of responsibility after cognitive offloading; and institutional climate focuses on the local norms and governance arrangements that shape how the other three are experienced. Their overlap is therefore substantive but not duplicative.
